# Flame-made nanoparticles for magnetic hyperthermia and MRI in colorectal cancer theranostics†

**DOI:** 10.1039/d5na00603a

**Published:** 2025-07-16

**Authors:** Yuming Zhang, Christina Paraskeva, Qianying Chen, Anano Maisuradze, Shaquib Rahman Ansari, Tapati Sarkar, Vasiliki Koliaraki, Alexandra Teleki

**Affiliations:** a Department of Pharmacy, Science for Life Laboratory, Uppsala University 75123 Uppsala Sweden alexandra.teleki@scilifelab.uu.se; b Institute for Fundamental Biomedical Research, Biomedical Sciences Research Center ‘Alexander Fleming’ 16672 Vari Greece; c Department of Materials Science and Engineering, Uppsala University Box 35 75103 Uppsala Sweden

## Abstract

Magnetic hyperthermia therapy using superparamagnetic iron oxide nanoparticles (SPIONs) offers a promising strategy for treating cancers resistant to chemo- and radiotherapy. However, oral delivery of SPIONs for localized treatment of gastrointestinal cancers has not been widely explored. Here, we report the development of methoxy polyethylene glycol (mPEG) functionalized SPIONs (mPEG-Mn_0.6_Zn_0.4_Fe_2_O_4_) engineered for oral administration with combined theranostic functionalities for magnetic hyperthermia treatment and magnetic resonance imaging (MRI) in colorectal cancer (CRC). The SPIONs achieved consistent heating performance in biorelevant colonic environments, exceeding a 5 °C temperature increase within 10 min under an alternating magnetic field (AMF). They also demonstrated superior *r*_2_ relaxivity compared to γ-Fe_2_O_3_, highlighting their potential as effective *T*_2_ MRI contrast agents. *In vitro* studies using CRC SW480 and Caco-2 cell lines assessed nanoparticle cytotoxicity, cellular uptake, and magnetic hyperthermia efficacy in both upright and inverted cell culture configurations. Magnetic hyperthermia induced significant CRC cell death *in vitro*, particularly in upright configurations, attributed to enhanced localized heating caused by nanoparticle sedimentation and enhanced SPION contact with cell surfaces. This emphasizes the importance of *in vitro* experimental parameters such as cell line, configuration, and AMF exposure time for systematic optimization of theranostic SPIONs during preclinical development. Finally, *in vivo* studies using a colorectal tumor xenograft mouse model demonstrated a marked therapeutic effect of magnetic hyperthermia by intratumorally injected SPIONs. The tumor volume was reduced by 63% following a single 20-minute AMF exposure. These findings demonstrate the potential of mPEG-Mn_0.6_Zn_0.4_Fe_2_O_4_ nanoparticles as a promising platform for non-invasive, image-guided magnetic hyperthermia therapy in CRC theranostics.

## Abstract 2

Flame-made SPIONs functionalized with mPEG demonstrated theranostic efficacy *in vitro* and *in vivo*, enabling MRI-guided hyperthermia and colorectal tumor regression.

## Graphical abstract

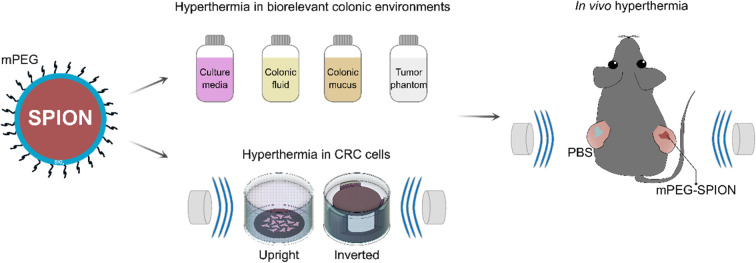

## Introduction

Colorectal cancer (CRC) is the third most common cancer and the second most common cause of cancer-related death in the world, imposing a substantial burden in terms of both human lives lost and medical resources.^1^ Effective treatment of CRC relies on early detection of the disease. While early-stage CRC can be detected *via* colonoscopy and be surgically excised, such interventions are invasive and often suffer from poor patient compliance.^2^ Later-stage CRC is typically removed by surgery followed by adjuvant chemotherapy, however, reoccurrence rates are high (∼20% for stage II and ∼30% for stage III CRC).^3^ Thus, developing a non-invasive theranostic agent that can locally act against CRC is urgently needed to complement current medical practices.

Nanotechnology has revolutionized cancer therapy by offering a spectrum of applications including drug delivery, treatment, diagnosis, and theranostics. Among different types of nanoparticles, superparamagnetic iron oxide nanoparticles (SPIONs) have emerged as exceptional theranostic agents due to their unique magnetic properties, high biocompatibility, and ease of surface functionalization.^4–6^ SPIONs consist of small (5 to 30 nm) iron oxide crystals (maghemite γ-Fe_2_O_3_, magnetite Fe_3_O_4_, or hematite α-Fe_2_O_3_) and a single magnetic domain, which leads to superparamagnetism, allowing them to get magnetized solely in the presence of an external magnetic field. Upon exposure to an external alternating magnetic field (AMF), the magnetic domain (Néel relaxation) or the particle itself (Brownian relaxation) aligns with the changing magnetic field direction and releases heat.^7^ This heat can be used in magnetic hyperthermia therapy, where it elevates the local tumor temperature to 43–46 °C.^8^ In fact, magnetic hyperthermia has been used clinically to treat glioblastoma through the injection of aminosilane-coated SPIONs directly to the tumor site followed by repeated AMF exposure.^9^ In addition to their therapeutic applications, SPIONs also serve as effective magnetic resonance imaging (MRI) contrast agents. By combining MRI contrast enhancement with magnetic hyperthermia properties, SPIONs offer a theranostic approach, enabling both diagnosis and localized thermal treatment of tumors.

Despite the numerous preclinical developments for cancer theranostics, the clinical potential of SPIONs has not yet been fully realized. Considerable research efforts have focused on synthesizing SPIONs with enhanced heating performance and improved MRI contrast efficacy. This can be achieved by fine-tuning the physicochemical properties of the SPIONs, such as composition, particle shape and size. The incorporation of dopants such as manganese or zinc improves both heating performance and MRI contrast. Ansari *et al.* demonstrated that Mn_0.25_Fe_2.75_O_4_ offers optimal hyperthermia performance with low cytotoxicity in CRC cell lines, outperforming undoped and Mg- or Zn-doped SPIONs.^10^ Similarly, Starsich *et al.* found that silica-coated Zn_0.4_Fe_2.6_O_4_, and Gd_0.225_Zn_0.4_Fe_2.375_O_4_ exhibit high *r*_2_ relaxivity and saturation magnetization,^11,12^ while Jang *et al.* reported that Mn_0.6_Zn_0.4_Fe_2_O_4_ shows the highest saturation magnetization and *r*_2_ relaxivity among Zn-doped manganese ferrites.^13^ These studies have systematically evaluated doped ferrites, reporting specific values for SPION magnetization and heating efficiency, but direct comparisons are challenging due to differences in experimental conditions, and the lack of measurements in biorelevant environments halt their clinical translation.

**Fig. 1:**
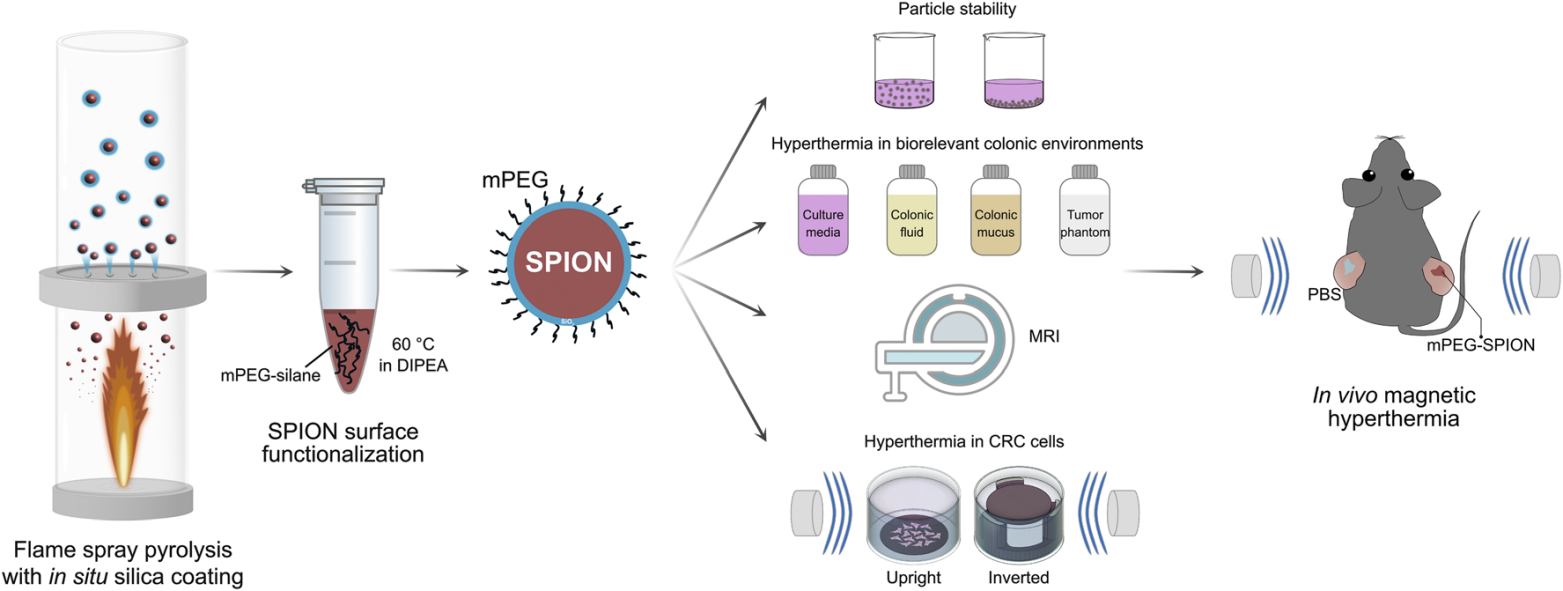
Schematic illustration of the synthesis, the *in vitro* and *in vivo* evaluation of PEGylated superparamagnetic iron oxide nanoparticles (SPIONs) for colorectal cancer (CRC) theranostics. Silica-coated SPIONs were synthesized using flame spray pyrolysis and subsequently functionalized with methoxy-polyethylene glycol silane (mPEG-silane) in *N*,*N*-diisopropylethylamine (DIPEA) to yield PEGylated SPIONs (mPEG-SPIONs). The mPEG-SPIONs were characterized in biorelevant colonic conditions, including assessments of colloidal stability, magnetic heating performance, contrast enhancement in magnetic resonance imaging (MRI), and magnetic hyperthermia-induced cytotoxicity in both upright (left) and inverted (right) cell culture configurations. Finally, the therapeutic efficacy of mPEG-SPIONs was evaluated in a CRC xenograft mouse model. Tumors were intratumorally injected with an mPEG-SPION suspension and subsequently exposed to an alternating magnetic field (AMF) to induce localized magnetic hyperthermia.

Especially oral administration of SPIONs remains poorly explored, despite its high patient compliance and convenience. SPIONs intended for CRC theranostics can be delivered in colon-targeted delivery platforms to enable their controlled local release.^14,15^ However, upon release, the complex luminal contents of the colon and the surrounding microenvironment such as the mucus barrier can promote nanoparticle aggregation, potentially compromising their magnetic heating efficiency.^6,17^ In this study, we synthesized silica-coated SPIONs with different dopants (Mn^2+^, Zn^2+^ and Gd^3+^) using flame spray pyrolysis (FSP) (Fig. 1). The nanoparticle surface was functionalized with methoxy polyethylene glycol (mPEG) to provide steric stabilization in colonic fluids. Finally, we demonstrated the diagnostic potential of the mPEG-Mn_0.6_Zn_0.4_Fe_2_O_4_ as an MRI contrast agent, highlighting its theranostic capabilities. The heating efficiency was evaluated in biorelevant colon environments and using both upright and inverted cell culture configurations. While inverted setups have been used for studying the impact of nanoparticle sedimentation on cellular uptake and cytotoxicity,^18–23^ their application in *in vitro* magnetic hyperthermia assessment remains unexplored. To our knowledge, this is the first study evaluating magnetic hyperthermia outcomes across different cell culture configurations. To support clinical translation, we further performed *in vivo* magnetic hyperthermia in a colorectal tumor xenograft mouse model.

## Materials and methods

### Flame synthesis of nanoparticles and surface functionalization

Silica-coated SPIONs were synthesized using an *in situ* flame spray pyrolysis (FSP) coating reactor as described previously.^24^ In brief, precursor solutions for γ-Fe_2_O_3_ Mn_0.25_Fe_2.75_O_4_, Zn_0.4_Fe_2_O_4_, Mn_0.6_Zn_0.4_Fe_2_O_4_ and Gd_0.225_Zn_0.4_Fe_2.375_O_4_ were prepared by dissolving manganese(ii) nitrate tetrahydrate (Sigma-Aldrich, purity 97%), zinc nitrate hexahydrate (Sigma-Aldrich, purity 98%), iron(iii) nitrate nonahydrate (Sigma-Aldrich, purity 98%) and gadolinium(iii) nitrate hexahydrate (Sigma-Aldrich, purity 99.99%) according to their molar ratio in a 1 : 1 (v/v) mixture of 2-ethylhexanoic acid (Sigma-Aldrich; 99%) and ethanol (VWR, >99.7%). The total metal concentration in the precursor solutions was adjusted to 0.34 M for all particle compositions. The precursor solution was stirred for at least 1 hour at room temperature to ensure complete dissolution. After that, it was fed through the center capillary of the FSP reactor at 5 mL min^−1^ for γ-Fe_2_O_3_, 3.5 mL min^−1^ for Mn_0.25_Fe_2.75_O_4_, 4 mL min^−1^ for Zn_0.4_Fe_2_O_4_, 4 mL min^−1^ for Mn_0.6_Zn_0.4_Fe_2_O_4_ and 5 mL min^−1^ for Gd_0.225_Zn_0.4_Fe_2.375_O_4_. The precursor spray was dispersed by 5 L min^−1^ O_2_ (Linde AGA Gas AB, Sweden, >99.5%), and ignited by a premixed CH_4_/O_2_ (1.5/3.2 L min^−1^) flame (pressure drop between 1.4 to 1.6 bar).

The FSP reactor was enclosed by a 20 cm long quartz glass tube and sheathed with 40 L min^−1^ O_2_ gas fed through the outermost sinter metal plate. A torus metal ring with 16 radial equally spaced openings was placed on top of the quartz glass tube, and the reactor was enclosed on top with another 30 cm long quartz tube.^24^ A saturated gas stream of N_2_ carrying hexamethyldisiloxane (HMDSO; Sigma-Aldrich, purity ≥ 99%) vapor was swirl injected through the torus ring with additional N_2_ at 15 L min^−1^ into the reactor. The silica coating precursor, HMDSO, was contained in a bubbler at 10 °C. The N_2_ flow rate through the HMDSO bubbler was adjusted to obtain 23% (w/w) SiO_2_ in the final product powder (0.34 L min^−1^ for γ-Fe_2_O_3_, 0.23 L min^−1^ for Mn_0.25_Fe_2.75_O_4_, 0.27 L min^−1^ for Zn_0.4_Fe_2_O_4_, 0.27 L min^−1^ for Mn_0.6_Zn_0.4_Fe_2_O_4_ and 0.37 L min^−1^ for Gd_0.225_Zn_0.4_Fe_2.375_O_4_). The product powders were collected on a glass fiber filter (Albert LabScience, Germany) placed above the FSP reactor with the aid of a Mink MM 1144 BV vacuum pump (Busch, Sweden). The silica-coated SPIONs are referred to as γ-Fe_2_O_3_, Mn_0.25_Fe_2.75_O_4_, Zn_0.4_Fe_2_O_4_, Mn_0.6_Zn_0.4_Fe_2_O_4_ and Gd_0.225_Zn_0.4_Fe_2.375_O_4_ in the following.

The surface of selected particles (γ-Fe_2_O_3_ and Mn_0.6_Zn_0.4_Fe_2_O_4_) was functionalized with methoxy polyethylene glycol (mPEG). *N*,*N*-Diisopropylethylamine (DIPEA) was used as reaction solvent and catalyst. mPEG-silane (5 kDa, 15 mg; Sigma-Aldrich) was added to γ-Fe_2_O_3_ or Mn_0.6_Zn_0.4_Fe_2_O_4_ nanoparticles (20 mg) and the mixture was sonicated in 10 mL of DIPEA for 5 minutes to obtain a nanoparticle–PEG slurry. The reaction was continued overnight at 60 °C under vigorous shaking. The final product was washed three times with methanol and dried in a vacuum oven at 60 °C for at least 2 hours.

### Nanoparticle characterization

The crystalline structure of as-produced nanoparticles was determined by X-ray diffraction (XRD; D2 PHASER, Bruker, Germany) at a step size of 0.06° between 20 and 80° 2*θ* at 1 s per step. The crystallite size of SPIONs was determined from the XRD patterns using the DIFFRAC.SUITE software (Bruker, Germany). The nanoparticles were degassed for at least 3 hours at 120 °C under a nitrogen flow, after which the specific surface area of the particles was measured by nitrogen adsorption (Brunauer–Emmett–Teller method, BET) at −196 °C on a TriStar II Plus system (Micromeritics, USA). The surface chemical groups of the nanoparticles were characterized by attenuated total reflectance-Fourier transform infrared spectroscopy (ATR-FTIR) from 400 cm^−1^ to 4000 cm^−1^ (FTIR; Alpha II, Bruker, USA).

Room temperature magnetization hysteresis loops of silica-coated γ-Fe_2_O_3_, Mn_0.25_Fe_2.75_O_4_, Zn_0.4_Fe_2.6_O_4_, Mn_0.6_Zn_0.4_Fe_2_O_4_ and Gd_0.225_Zn_0.4_Fe_2.375_O_4_ were recorded using a vibrating sample magnetometer (Lake Shore 7404 VSM). The magnetization *versus* magnetic field was measured in the field range of ±10 000 Oe. The saturation magnetization (*M*_s_) and coercivity (*H*_c_) were obtained from the magnetization curves. The mPEG content on the functionalized γ-Fe_2_O_3_ and Mn_0.6_Zn_0.4_Fe_2_O_4_ was determined by thermogravimetric analysis (TGA; TGA 500, TA Instrument, Germany) with a heating rate of 10 °C min^−1^ from 25 to 900 °C under nitrogen flow. The TGA data were normalized to the weight loss at 120 °C to account for adsorbed water on the samples.^25^ The surface coverage density of mPEG (*σ*), mPEG footprint (FP), distance between anchored mPEG chains on the surface (*D*), and the unperturbed layer thickness were calculated according to the literature.^26^

The morphologies of mPEGylated γ-Fe_2_O_3_ and Mn_0.6_Zn_0.4_Fe_2_O_4_ were imaged using transmission electron microscopy (TEM) with a JEM-2100F (Jeol Ltd., Japan) and a Schottky-type field emission gun operating at 200 kV. For the imaging, a nanoparticle suspension at 0.01 mg mL^−1^ was prepared by cuphorn ultrasonication (Sonics, USA) in 99.5% ethanol. Then, approximately a 10 μL nanoparticle suspension was placed on a Formvar–carbon 300-mesh copper grid (Delta Microscopies, France) and the ethanol was allowed to evaporate.

The hydrodynamic diameter of particles in various aqueous environments (1 mg mL^−1^) was measured by dynamic light scattering (DLS) at 37 °C, and the data were collected from the backscattered light (Litesizer 500, Anton Paar, Austria). The zeta potential was measured in MilliQ water (1 mg mL^−1^) at 37 °C by electrophoretic light scattering (ELS) using the same instrument. The samples used for DLS and ELS were diluted from a stock suspension of SPIONs (10 mg mL^−1^ in water) prepared by sonication (Vibra-Cell, Sonics & Materials, Inc., USA). Samples were sonicated at 90% amplitude in cooling water for 5 minutes with vortexing every minute. The particle stock was diluted to 1 mg mL^−1^ in six different media: MilliQ water, cell culture medium, cell culture medium supplied with 10% (v/v) fetal bovine serum (cell culture medium + FBS), fasted state simulated colonic fluid (FaSSCoF, Biorelevant, UK), phosphate-buffered saline (PBS, pH 7.4) and 50 mM 2-(*N*-morpholino)ethanesulfonic acid (MES) buffer (pH 6.5). The cell culture medium consisted of Dulbecco's modified Eagle medium with 1% (v/v) l-glutamine, 1% (v/v) nonessential amino acids, and 1% (v/v) penicillin–streptomycin (all from ThermoFisher Scientific). These six particle suspensions were incubated at 37 °C for up to 24 hours. To assess mPEG-SPION stability in the six environments, hydrodynamic diameter was measured at 10 min, and then after 1, 4, and 24 h. Sedimented particles were gently re-dispersed by pipetting prior to DLS measurements.

### Heat dissipation measurements

Hyperthermia measurements were performed using an oscillating magnetic field apparatus (MagneTherm, nanoTherics Ltd., United Kingdom). mPEGylated nanoparticles were characterized in (i) MilliQ water, (ii) fasted state colonic fluid (FaSSCoF), (iii) porcine artificial colonic mucus (PACM), (iv) a 2% agar tumor tissue phantom, and (v) complete cell culture medium (Dulbecco's modified Eagle medium supplied with 10% fetal bovine serum and 1% non-essential amino acid and 1% penicillin–streptomycin). PACM was prepared according to a published protocol.^27^ A stock suspension (10 mg mL^−1^ in water) of mPEG-SPIONs was prepared by sonication at 90% amplitude for 5 min, with vortexing between each minute. To prepare nanoparticle suspension in water, FaSSCoF, cell culture media or liquified warm agar, stock suspension were diluted and vortexed with these media at a concentration of 1 mg mL^−1^. The aqueous nanoparticle suspension used for the *in vivo* study was measured undiluted at 10 mg mL^−1^. To prepare a homogeneous mPEG-SPION and PACM mixture, the nanoparticle–PACM mixture (1 mg mL^−1^) was repetitively extruded through a syringe with a 1.2 × 50 mm needle. The thermal dissipation of 500 μL nanoparticle suspensions in water or biorelevant environments were measured. All samples were placed in a 15 mL falcon tube and preheated to 37 °C, after which the tube was placed in the center of a 9-winding coil (diameter = 44 mm) with a 37 °C water jacket surrounding it. After temperature equilibration, an AMF at 592.3 kHz frequency (*f*) and 14 mT amplitude (*H*) was applied for 10 min. This magnetic field is below the clinically acceptable threshold reported as the Brezovich limit.^28^ Temperature changes of the samples were continuously recorded using a fiber optic probe (OSENSA, Canada). The temperature increase (Δ*T*) was calculated according to eqn (1), and the specific absorption rate (SAR) as in eqn (2):

1 Δ*T* = *T*_10 min_ − *T*_0 min_

2
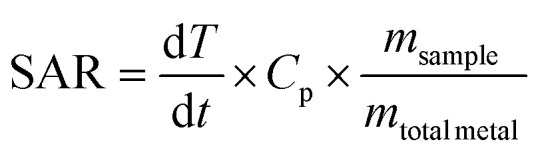
where 
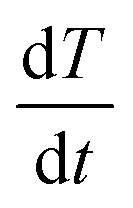
 is the maximum slope of the heating curve; *C*_p_ is the heat capacity of water; *m*_sample_ is the mass of the sample which is estimated to be equivalent to the mass of the water in each sample; *m*_total metal_ is the mass of total metal in each sample.

### Magnetic resonance imaging

Magnetic resonance imaging (MRI) was carried out using a spin echo (SE) sequence on a BioSpec 94/30 (Bruker BioSpin MRI GmbH) at 9.4 T. The mPEGylated γ-Fe_2_O_3_ and Mn_0.6_Zn_0.4_Fe_2_O_4_ were dispersed in an agar suspension (0.75%) with a series of total metal concentrations of 0.025, 0.05, 0.1, 0.2, and 0.4 mM. For *T*_2_-weighted images, repetition time (TR) was 6000 ms and echo times (TEs) were 4, 10, 16, 28, 40, 52, 64, and 76 ms. Relaxation rates (*R*_2_) were obtained by exponential fitting using the MRI signal and TE in eqn (3):

3 *I*(*TE*) = *I*(0) × e^−TE×*R*_2_^

where *I*(TE) is the MRI signal intensity at a specific echo time and *I*(0) is the MRI signal at zero echo time.

Relaxivity (*r*_2_) was attained by normalizing the relaxation rates according to eqn (4):4
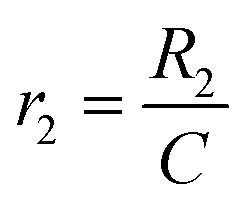
where *C* is the metal concentration, and *R*_2_ is the relaxation rate.

### Cell culture

Human colorectal adenocarcinoma cell lines Caco-2 (originally obtained from American Type Culture Collection) and SW480 (ATCC CCL-228) were cultured in complete cell culture medium. The cells were maintained at 37 °C in an incubator (90 to 100% humidity) supplied with 10% CO_2_. The cells used for all experiments were obtained within 10 passages after thawing. Mycoplasma contamination tests were conducted on each newly thawed batch of cells.

### Cytotoxicity of nanoparticles

The cytotoxicity of as-prepared and PEGylated γ-Fe_2_O_3_ or Mn_0.6_Zn_0.4_Fe_2_O_4_ was evaluated in SW480 and Caco-2 cells. Cells were plated in 96-well tissue culture plates at a density of 155 000 cells per cm^2^ in 200 μL of culture medium 24 h prior to nanoparticle treatment. The cells were then exposed to freshly prepared nanoparticle suspensions at various concentrations (0.1, 0.2, 0.4, 0.6, 0.8, 1 mg mL^−1^) for 24 hours before assessing cell viability. Cell culture medium and 0.22% (v/v) sodium dodecyl sulfate were used as negative and positive controls, respectively. Cell viability was assessed with CellTiter-Glo luminescent assay (Promega, USA) according to the manufacturer's protocol.

### Nanoparticle cellular uptake

The cellular uptake of mPEG-Mn_0.6_Zn_0.4_Fe_2_O_4_ was studied in both upright and inverted cell culture configurations. 13 mm round plastic coverslips (Nunc™ Thermanox™ Coverslips, ThermoFisher Scientific) were placed in each well of a 24-well plate (well diameter: 13 mm) and cells were seeded at a density of 155 000 cells per cm^2^ for 24 h. For the upright configuration, the coverslips were then transferred to a new 24-well plate with the cells facing upwards. In the inverted configuration, the coverslips were placed on customized polycarbonate inserts (cells facing downwards) and the insert fitted into a 24-well plate (Fig. 1). The inserts were machined from polycarbonate stock on a Nomad desktop CNC mill (Carbide 3D, USA) and the toolpath was generated using Autodesk Fusion (Fig. S1 and S2†). 1.2 mL of cell culture medium with 0.4 mg mL^−1^ of mPEG-SPIONs was added to both configurations and incubated for 2 h. The hydrodynamic diameter of the particle suspensions in cell culture medium was measured before each experiment to verify suspension stability (*d*_DLS_ < 200 nm).

After incubation, the cells were washed three times with warm PBS to remove surface-bound nanoparticles. Subsequently, the cells were trypsinized, collected, and counted (Countess automated cell counter; ThermoFisher Scientific). The cells were then lysed in 500 μL 1 M NaOH under vigorous shaking at 60 °C overnight and 1 mL of 37% HCl was added the next day. These cell lyses solutions were diluted 10–15 times with 5% HNO_3_ and filtered (0.2 μm) before measuring the Fe concentration by inductively coupled plasma-optical emission spectroscopy (ICP-OES; Avio 200 Scott/Cross-Flow Configuration, PerkinElmer, USA). A calibration curve was obtained with a Multielement Calibration Standard (CPAchem, Bulgaria).

### *In vitro* magnetic hyperthermia

Magnetic hyperthermia treatments were conducted for two cycles with both configurations (inverted and upright) and cell lines (Caco-2 and SW480) by adding mPEG-Mn_0.6_Zn_0.4_Fe_2_O_4_ (0.4 mg mL^−1^) to the media. The cell culture dish was placed in the center of the water-cooled coil and exposed to an AMF (*f* = 592.3 kHz, *H* = 14 mT) for 60 minutes. The cells were then incubated in a humidified incubator supplied with 10% CO_2_ for 24 hours before the AMF exposure was repeated another 60 minutes. Cells incubated with medium, SPIONs alone, or AMF alone were used as controls throughout the two treatment cycles. Cell death was assessed using the LDH-Glo Cytotoxicity Assay (Promega, USA) immediately after each of the two AMF exposures.

### MC38 xenograft mouse model

Four-month-old male and female mice on the C57Bl/6 background were used for *in vivo* experiments. Mice were housed in the animal house facility of the Biomedical Sciences Research Center Alexander Fleming under specific pathogen-free conditions with controlled temperature (22 ± 2 °C), humidity (55 ± 10%), and a 12 hour light/dark cycle. All animal experiments were approved by the Institutional Animal Care and Use Committee of BSRC Fleming (protocol number: 1175208) and conducted in accordance with European and national guidelines for the care and use of laboratory animals.

13 wild-type mice were injected subcutaneously with 500 000 MC38 cells per injection on both flanks. Tumor growth was monitored every two days using a digital caliper. Tumors reached approximately a size of 7–9 mm, 7–9 days after cell injections.

### *In vivo* magnetic hyperthermia treatment

Mice (*n* = 7; males = 3, females = 4) were weighed and injected with an anesthesia cocktail (200 mg kg^−1^ ketamine, 15 mg kg^−1^ xylazine and 0.05 mg kg^−1^ atropine) at a dose of 5 μL g^−1^ of body weight prior to mPEG-SPION suspension injection. Once anesthetized, each mouse received an intratumoral injection of 200 μL of either a 10 mg mL^−1^ PEG-SPION water suspension or pure PBS control into one of the tumors located on the back, respectively. Tumor height and width were measured before injection using a digital caliper. Following injection, the mouse was transferred to a 37 °C water jacket and positioned at the center of a 9-turn coil to ensure maximum exposure to the AMF (*H* = 14 mT, *f* = 590.6 kHz) for 20 minutes. In one exception, AMF exposure was terminated at 14 minutes due to premature awakening of the animal. The position of each mouse was adjusted to center the tumor within the coil. Tumor temperature was monitored using an infrared thermal camera (Fluke Ti480 Pro, Fluke Europe, The Netherlands). Mice were then housed for another 2 days before sacrificing. A separate control group (*n* = 6; males = 4, females = 2), injected with either mPEG-SPION suspension or PBS into one of the tumors, underwent the same experimental procedure, excluding AMF exposure.

The length and width of the tumors were measured on the day of sacrifice. The volume of the tumor (*V*) was calculated with eqn (5):5
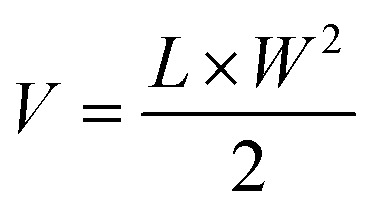
where *L* is the tumor length and *W* is the tumor width.

Tumors were dissected and washed in PBS followed by fixation in 10% neutral buffered formalin for at least 5 hours at 4 °C. Tumors were then washed again in PBS, processed in the Spin Tissue Processor (Leica TP1020) and embedded in paraffin. Tissue sections were obtained using a microtome (SLEE medical) at 4 μm and stained with hematoxylin and eosin using the Leica ST5010 XL autostainer. H&E-stained tissue sections were imaged using an Olympus Slide Scanner VS200 (20× lens) and the OlyVIA (Ver.2.9.1) software.

## Results and discussion

### Physicochemical and magnetic properties of SPIONs

Silica-coated SPIONs were manufactured by FSP. This technique is highly scalable and reproducible with fine control of size and composition of the nanoparticles. The ability of FSP to produce SPIONs at a high production rate (10 kg h^−1^) makes it suitable for industrial manufacturing.^10,29^ Coating with SiO_2_ was performed *in situ* during FSP synthesis to improve particle biocompatibility, dispersibility in aqueous media, and to facilitate subsequent surface functionalization.^24^ Moreover, SiO_2_ coating increases the saturation magnetization compared to uncoated SPIONs, which typically correlates positively with heating performance in magnetic hyperthermia.^11,12^

The aim of our study was to produce SiO_2_ coated-SPIONs with a heating performance suitable for magnetic hyperthermia and enhanced MRI contrast for CRC theranostics. Therefore, we produced doped SPIONs and evaluated their heating performance to identify the composition with optimal heating performance in a biorelevant environment. In total, four doped nanoparticles—Mn_0.25_Fe_2.75_O_4_, Zn_0.4_Fe_2.6_O_4_, Mn_0.6_Zn_0.4_Fe_2_O_4_ and Gd_0.225_Zn_0.4_Fe_2.375_O_4_—along with γ-Fe_2_O_3_ as a benchmark, were synthesized and evaluated for their physicochemical properties and heating performance. FSP process parameters (*i.e.* precursor flow rate) were selected to yield nanoparticles with a particle core size within the superparamagnetic domain (10–30 nm).^10,30,31^ The optimal heating performance of flame-made ferrites has been reported to be in the 15–18 nm range.^10^ Thus, in our study, the crystallite size of all particles was controlled to be near 15 nm for optimal hyperthermia outcomes.

**Fig. 2:**
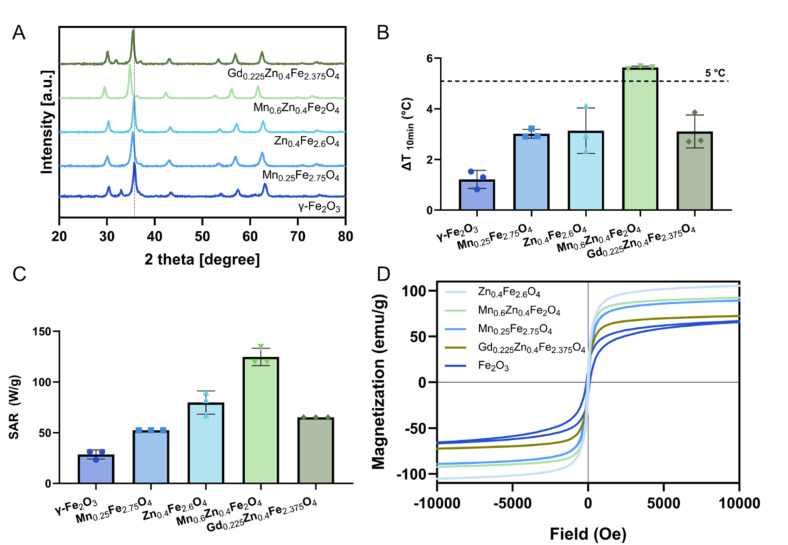
Characterization of silica-coated SPIONs with different core compositions. (A) XRD pattern of silica-coated γ-Fe_2_O_3_, Mn_0.25_Fe_2.75_O_4_, Zn_0.4_Fe_2.6_O_4_, Mn_0.6_Zn_0.4_Fe_2_O_4_ and Gd_0.225_Zn_0.4_Fe_2.375_O_4_. The dashed line marks the (311) plane of the maghemite crystal structure. (B) Temperature change (Δ*T*), and (C) specific absorption rate (SAR) of silica-coated doped ferrites in water suspension after 10 min of AMF exposure (*f* = 592.3 kHz, *H* = 14 mT). Data are expressed as mean ± SD (*n* = 3). The dashed line in (B) represents Δ*T* = 5 °C, the temperature increase commonly recommended for hyperthermia treatment. (D) Magnetic field dependence of magnetization at 27 °C of silica-coated γ-Fe_2_O_3_, Mn_0.25_Fe_2.75_O_4,_ Zn_0.4_Fe_2.6_O_4_, Mn_0.6_Zn_0.4_Fe_2_O_4_ and Gd_0.225_Zn_0.4_Fe_2.375_O_4_.

Fig. 2A shows the X-ray diffraction (XRD) patterns of the as-produced nanoparticles and Table 1 lists their physicochemical properties. The prominent peaks of γ-Fe_2_O_3_ at 2*θ* = 30.3°, 35.8°, 43.4°, 53.8°, 57.4° and 63.1° correspond to the spinel cubic structure of maghemite.^24,32^ A small peak at 2*θ* = 32.8° was also identified, indicating the presence of α-Fe_2_O_3_ (hematite, (104) plane), in agreement with literature for flame synthesis in an enclosed reactor.^24^ The diffraction peaks shifted towards a lower 2*θ* angle upon doping (with Mn^2+^, Zn^2+^ and Gd^3+^, indicated by the dashed line at the (311) plane in Fig. 2A) and the (104) plane of hematite could no longer be discerned in Mn_0.25_Fe_2.75_O_4_, Zn_0.4_Fe_2.6_O_4_ and Mn_0.6_Zn_0.4_Fe_2_O_4_. This indicates the successful incorporation of the dopants in the iron oxide crystal lattice and the formation of a magnetite spinel ferrite structure.^33^ For Gd_0.225_Zn_0.4_Fe_2.375_O_4_, a small peak appears at 2*θ* = 31.9° in agreement with such particles previously made by FSP.^11^

**Table 1:** Crystallite size (*d*_XRD_), specific surface area, hydrodynamic diameter and zeta potential of the flame-made, SiO_2_-coated SPIONs. All data expressed as mean ± SD (*n* = 3)

Particle compositions	*d*_XRD_ (nm)	Specific surface area (m^2^ g^−1^)	Hydrodynamic diameter^a^ (nm)	Zeta potential (mV)
γ-Fe_2_O_3_	16.2	46.5	185 ± 5	−53 ± 2
Mn_0.25_Fe_2.75_O_4_	13.0	52.1	176 ± 2	−37 ± 1
Zn_0.4_Fe_2.6_O_4_	16.0	47.1	174 ± 5	−35 ± 2
Mn_0.6_Zn_0.4_Fe_2_O_4_	15.5	52.0	184 ± 8	−34 ± 2
Gd_0.225_Zn_0.4_Fe_2.375_O_4_	15.8	38.7	187 ± 4	−21 ± 1

a Hydrodynamic diameter was measured at 1 mg mL^−1^ in H_2_O.

The crystallite size of all nanoparticles was about the targeted 15 nm (Table 1). The presence of silica in the flame-made particles could not be discerned in the XRD patterns; however, it was indicated by the negative zeta potential of the particles. This negative surface charge enhanced the dispersibility of SPIONs in water, resulting in a small hydrodynamic diameter (∼180 nm) at a relatively high SPION particle concentration (1 mg mL^−1^). In contrast, the literature reports uncoated SPIONs to have significantly larger hydrodynamic diameters, >2000 nm, even at low concentrations (0.1 mg mL^−1^) in water.^34^

Fig. 2B shows the heating performance of the nanoparticles in water under an AMF. All doped ferrite nanoparticles showed superior heating performance compared to pure γ-Fe_2_O_3_ in terms of both Δ*T* (Fig. 2B) and specific absorption rate (SAR) (Fig. 2C), with Mn_0.6_Zn_0.4_Fe_2_O_4_ achieving the highest values among the tested compositions. The enhanced heating performance of doped-SPIONs can be attributed to the substitution of dopant cation (Mn^2+^, Zn^2+^, Gd^3+^) at either A (the tetrahedral site) or B (the octahedral site) in the cubic spinel structure of magnetite. This occurs *via* two possible mechanisms: (i) weakening of the antiferromagnetic interaction by occupying the non-magnetic A site with dopants, allowing the magnetic B site to dominate, or (ii) incorporation of cations with a high spin state at the magnetic B site, thereby increasing the overall magnetic moment of the material.^13,35–37^

The heating performance of nanoparticles is governed by their size and magnetic properties such as the saturation magnetization, coercivity, and remanence.^10^Fig. 2D shows the magnetization of flame-made silica-coated SPIONs. All particles except γ-Fe_2_O_3_ displayed near zero hysteresis, confirming their superparamagnetic properties. A hysteresis loop was observed for γ-Fe_2_O_3_ (Fig. S3†) and could be attributed to the existence of a ferrimagnetic contribution from blocked particles.^24,38^ The saturation magnetization was much higher for Mn_0.6_Zn_0.4_Fe_2_O_4_ (94.8 emu g^−1^) compared to γ-Fe_2_O_3_ (70.4 emu g^−1^) as expected, which could explain its enhanced heating performance. Although Zn_0.4_Fe_2.6_O_4_ had the highest saturation magnetization among all tested SPIONs, its heating performance was inferior to that obtained for Mn_0.6_Zn_0.4_Fe_2_O_4_. This indicates that saturation magnetization alone does not fully account for heating efficiency. Instead, low coercivity has been shown to improve heating performance in flame-made silica-coated SPIONs, as reported by Starsich *et al.*^11^ As shown in Fig. S3,† Mn_0.6_Zn_0.4_Fe_2_O_4_ exhibited lower coercivity than Zn_0.4_Fe_2.6_O_4_, which could contribute to its superior thermal response under AMF.

### Nanoparticle surface mPEGylation

**Fig. 3:**
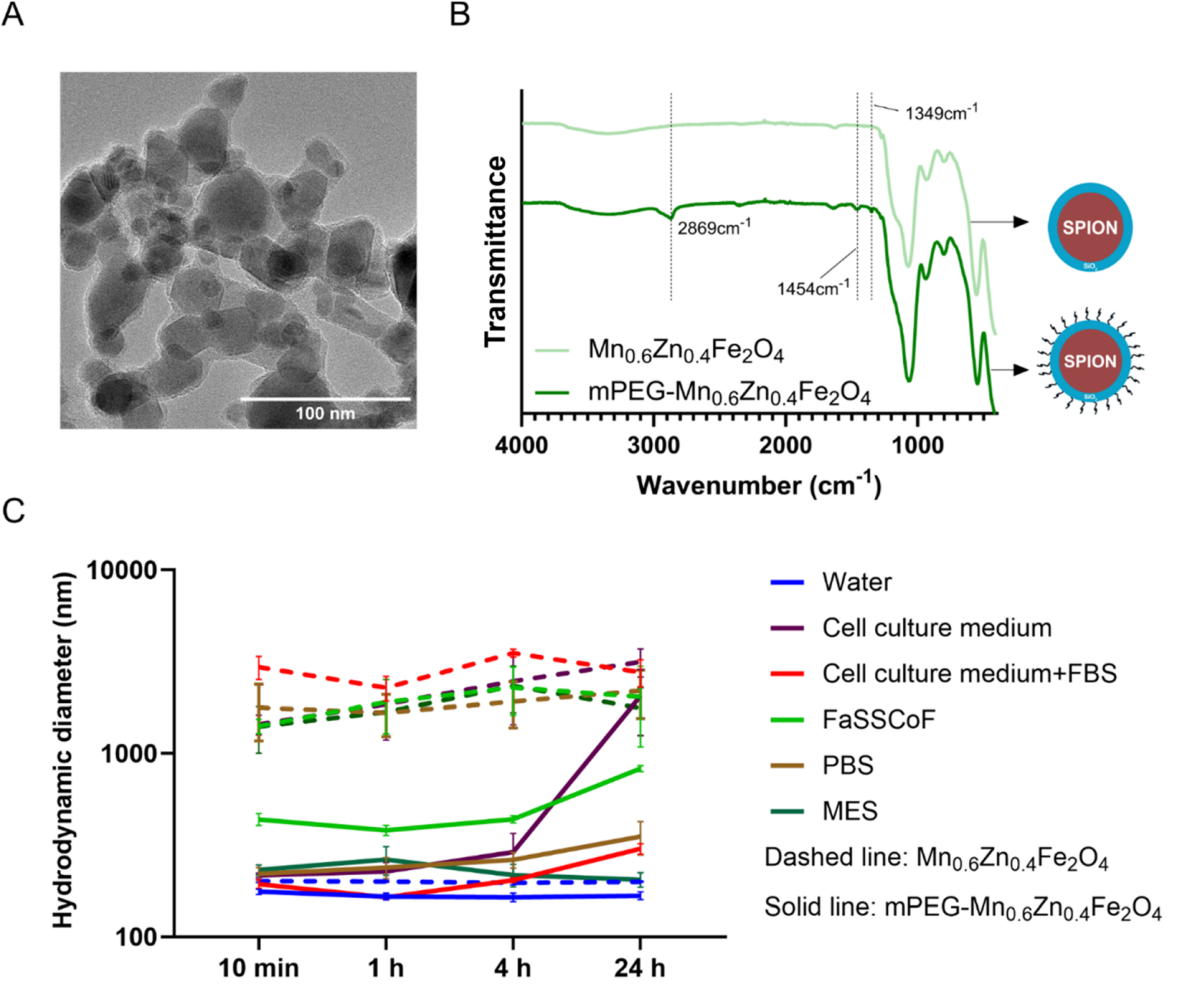
Characterization of the mPEGylated Mn_0.6_Zn_0.4_Fe_2_O_4_ and its colloidal stability in biorelevant biological fluids. (A) Transmission electron microscope image of the mPEG-Mn_0.6_Zn_0.4_Fe_2_O_4_. (B) FTIR spectra before (light green) and after surface PEGylation (dark green). (C) Characterization of particle suspension stability in biological fluids. Hydrodynamic diameter of Mn_0.6_Zn_0.4_Fe_2_O_4_ (1 mg mL^−1^) before (dashed lines) and after mPEGylation (solid lines) over 24 hours in water (blue), cell culture medium (dark red), cell culture medium with fetal bovine serum (FBS; bright red), simulated fasted-state colonic fluid (FaSSCoF; green), phosphate-buffered saline (PBS; brown), and 2-(*N*-morpholino)ethanesulfonic acid (MES; dark green). All data are expressed as mean ± SD (*n* = 3).

The Mn_0.6_Zn_0.4_Fe_2_O_4_ nanoparticles were selected for further *in vitro* evaluation due to their superior hyperthermia performance (Fig. 2B and C). Undoped silica-coated γ-Fe_2_O_3_ nanoparticles were also included to assess the impact of dopants in the cellular assays. The selected Mn_0.6_Zn_0.4_Fe_2_O_4_ and γ-Fe_2_O_3_ nanoparticles were functionalized with PEG after flame synthesis to improve their suspension stability in biological media. While the SiO_2_ coating effectively stabilizes SPION suspensions in water, it cannot prevent nanoparticle aggregation in biologically relevant fluids (Fig. 3 for mPEG-Mn_0.6_Zn_0.4_Fe_2_O_4_ and Fig. S6† for mPEG-Fe_2_O_3_). Thus, PEG with a methoxy end group (mPEG) was grafted to the silica surface *via* a covalent Si–O bond. This functionalization can be performed in a single synthesis step and typically results in a high density of PEG on the SPION surface.^39^ mPEG with a molecular weight of 5 kDa is reported optimal to reduce protein corona formation and was thus selected for our study.^40^Fig. 3A shows the TEM image of the SiO_2_-coated Mn_0.6_Zn_0.4_Fe_2_O_4_ after surface mPEGylation. A nano-thin layer of amorphous silica hermetically encapsulates the core nanoparticles, in agreement with the particle morphology reported for this synthesis method.^24^

The mPEGylation was confirmed by the appearance of bonds characteristic of mPEG in FTIR spectra (Fig. 3B). The bands at 2869, 1454 and 1349 cm^−1^ in the PEGylated SPIONs were associated with –CH_2_ stretching, –CH_3_ asymmetric bending and –C–O–C ether antisymmetric stretching, respectively.^41,42^ The zeta potential shifted to less negative values after PEGylation (Table S1†), which also indicates a successful surface functionalization with neutral PEG molecules on the negatively charged nanoparticle surface. PEG content, as determined by TGA, was comparable for both nanoparticle compositions: 11.0 ± 0.7% and 11.7 ± 1.3% for PEGylated γ-Fe_2_O_3_ and Mn_0.6_Zn_0.4_Fe_2_O_4_, respectively (Table S1†). A high surface coverage density of about 0.30 mPEG per nm^2^ was achieved for both SPION compositions, which is more than 48 times greater than the density required for a mushroom-to-brush regime transition for 5 kDa PEG.^43^ The distance between anchored PEG chains on the SPION surface was less than the Flory radius of 5 kDa PEG (6.0 nm). Thus, it can be assumed that a dense brush conformation of PEG chains formed on the SPION surface.^43^

A high PEG grafting density can provide SPIONs with steric stabilization and hydration repulsions in complex biological media. As shown in Fig. 3C, there was no significant change in hydrodynamic diameter after 24 hours in water for both SiO_2_-coated and mPEGylated nanoparticles. Silica-coated particles without mPEG surface functionalization rapidly agglomerated in all other media, evident by their large hydrodynamic diameters (>1000 nm). In contrast, the hydrodynamic diameter of mPEGylated Mn_0.6_Zn_0.4_Fe_2_O_4_ in cell culture medium was 200–300 nm during the first 4 hours of incubation but increased to >1000 nm after 24 hours. Notably, in the presence of fetal bovine serum (FBS), the hydrodynamic diameter of mPEGylated Mn_0.6_Zn_0.4_Fe_2_O_4_ remained stable at 150–200 nm throughout the entire 24 hour period, which could be attributed to the formation of a stabilizing protein corona.^44^ Particle suspensions of mPEGylated Mn_0.6_Zn_0.4_Fe_2_O_4_ were also stable in FaSSCoF, PBS and MES. The suspension stability of mPEGylated γ-Fe_2_O_3_ in biorelevant media was comparable to that observed for Mn_0.6_Zn_0.4_Fe_2_O_4_ (Fig. S6C†). Thus, surface PEGylation significantly improved the particle suspension stability in various biorelevant media, rendering them suitable for *in vitro* and *in vivo* evaluation for local magnetic hyperthermia therapy. Specifically, nanoparticles <200 nm, have been previously reported to be effective in passive tumor targeting.^45^

### Heating efficiency in biorelevant colonic environments

The heating efficiency of mPEGylated SPIONs was investigated in biorelevant environments encountered in the colon. The nanoparticles developed here are intended for local action at the colonic tumor site. They may therefore be exposed to colonic mucus and luminal fluid contents. The microenvironment surrounding the tumor can affect the heating performance of the SPIONs. In particular, higher surrounding viscosity limits the Brownian relaxation contribution to magnetic hyperthermia.^46^

**Fig. 4:**
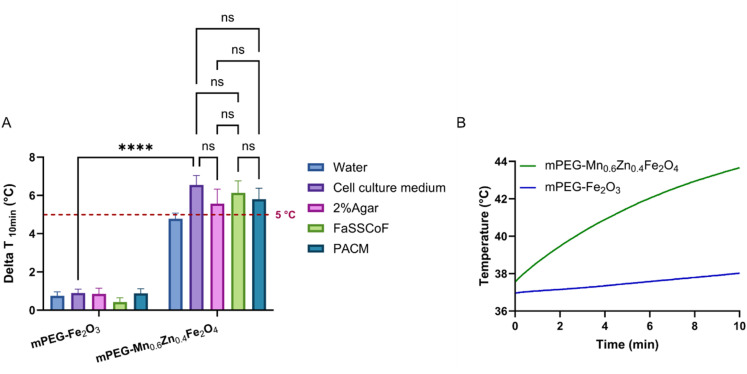
The heating performance of mPEG-Fe_2_O_3_ and mPEG-Mn_0.6_Zn_0.4_Fe_2_O_4_ in water and four biorelevant colonic environments. (A) Temperature change (Δ*T*) of mPEGylated γ-Fe_2_O_3_ and Mn_0.6_Zn_0.4_Fe_2_O_4_ in water (blue), cell culture medium (purple), 2% agar (representing tumor tissue structure; pink), simulated human fasted state colonic fluid (FaSSCoF; green) and porcine artificial colonic mucus (PACM; dark blue). The samples were exposed to an AMF (*f* = 592.3 kHz, *H* = 14 mT) for 10 min at 37 °C. The particle concentration was 1 mg mL^−1^. Data are expressed as mean ± SD (ns > 0.05, ****p ≤ 0.0001, *n* = 3). (B) Heating curve of mPEG-Mn_0.6_Zn_0.4_Fe_2_O_4_ (green) and mPEG-Fe_2_O_3_ (blue) during 10 min of AMF exposure.

The Δ*T* values generated by the SPIONs under AMF exposure were similar across all biorelevant colonic environments (Fig. 4A). Thus, the heat generation by the SPIONs can be attributed primarily to Néel's relaxation, *i.e.*, is not impacted by the viscosity of the surrounding environment. Furthermore, the heating efficiency in cell culture medium (with 10% FBS) was investigated for the subsequent *in vitro* cellular assays and no significant difference was measured compared to the biorelevant colonic environments. This supports the biorelevance of the subsequent *in vitro* assessment of magnetic hyperthermia with the SPIONs using colonic cancer cell lines. Mn_0.6_Zn_0.4_Fe_2_O_4_ had a significantly higher heating efficiency compared to γ-Fe_2_O_3_ (Fig. 4B), as expected from the magnetic properties of these nanoparticles (Fig. 2D).

### MRI contrast enhancement

**Fig. 5:**
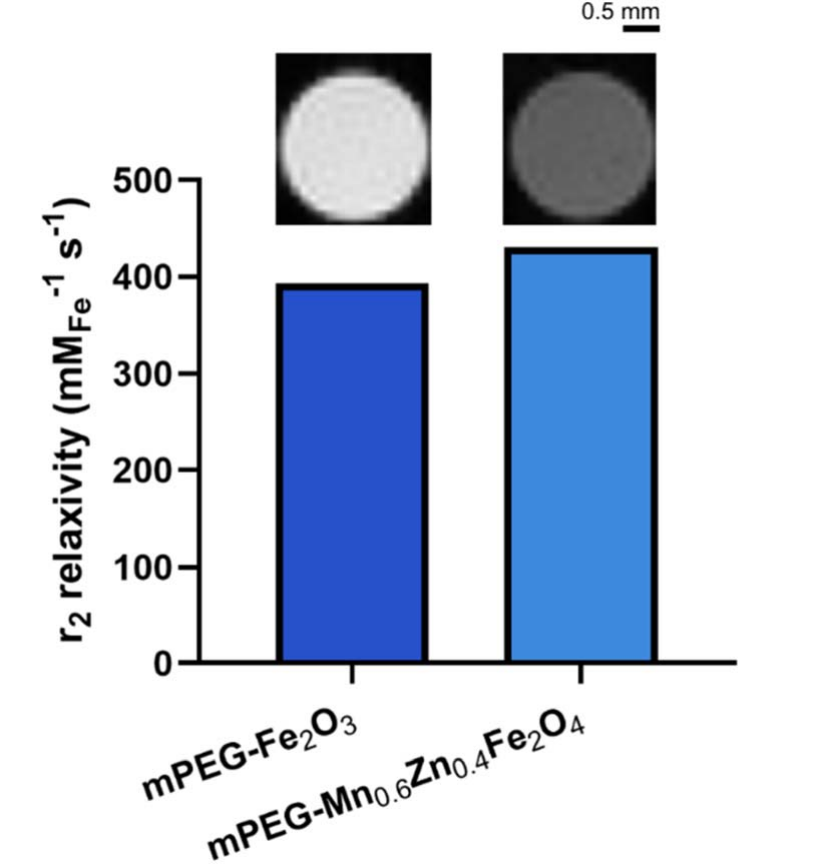
*r*_2_ relaxivity of mPEGylated γ-Fe_2_O_3_ and Mn_0.6_Zn_0.4_Fe_2_O_4_ measured at 9.4 T. The insets above the bars show the *T*_2_-weighted images of the corresponding particles.

The mPEG-Mn_0.6_Zn_0.4_Fe_2_O_4_ nanoparticles showed promising potential as magnetic hyperthermia agent in terms of physical stability in the colonic microenvironment (Fig. 3) and heating performance (Fig. 4). Precise localization of the nanoparticles *in vivo* is of importance to circumvent magnetic hyperthermia-induced death of healthy cells in the GIT in theranostic applications. To this end, we assessed the SPIONs for their contrast enhancement in MRI. Fig. 5 shows the *r*_2_ relaxivity of mPEG-Mn_0.6_Zn_0.4_Fe_2_O_4_ and mPEG-Fe_2_O_3_ dispersed in agar gels. The *r*_2_ relaxivity of mPEG-Mn_0.6_Zn_0.4_Fe_2_O_4_ and mPEG-Fe_2_O_3_ was 430 and 393 mM_Fe_^−1^ s^−1^, respectively, both more than twice as high as that of the commercial MRI contrast agent Resovist® (179 mM_Fe_^−1^ s^−1^).^47^ The higher *r*_2_ relaxivity of doped ferrite generated a stronger signal in *T*_2_-weighted images and thus the image appeared darker (Fig. 5, inset). The superior performance of Mn_0.6_Zn_0.4_Fe_2_O_4_ for negative contrast enhancement in MRI could be attributed to its higher saturation magnetization (Fig. 2D).^48^ Future studies should evaluate its *in vivo* performance as an MRI contrast agent.

Overall, the combination of excellent colloidal stability of mPEGylated Mn_0.6_Zn_0.4_Fe_2_O_4_ nanoparticles in biorelevant colonic media, outstanding magnetic heating efficiency, and strong MRI contrast enhancement, render these flame-made SPIONs suitable as theranostic agents for CRC treatment.

### Nanoparticle cytotoxicity and cellular uptake

**Fig. 6:**
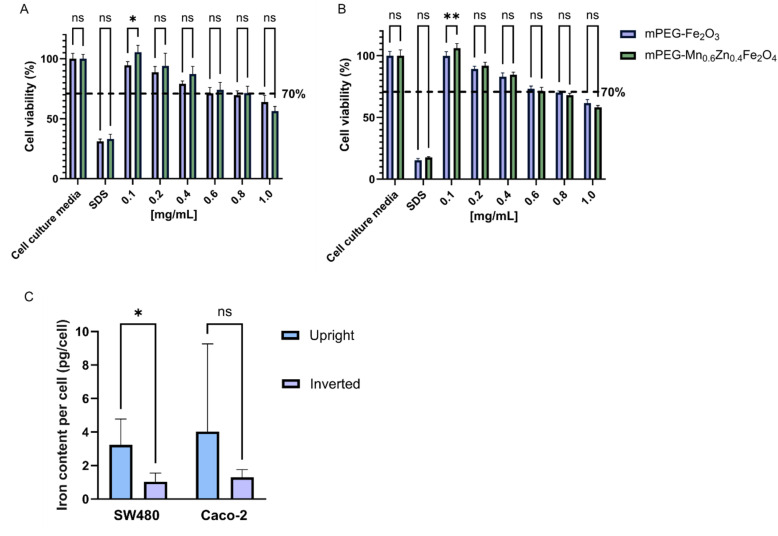
Nanoparticle cytotoxicity and cellular uptake of mPEGylated SPIONs in SW480 and Caco-2 cell lines. Viability of (A) SW480 and (B) Caco-2 cells exposed to mPEGylated SPIONs in concentration ranges from 0.1–1.0 mg mL^−1^ for 24 h in cell culture media. Cell culture medium and 0.22% (v/v) sodium dodecyl sulfate were used as negative and positive controls, respectively. The black dashed lines represent 70% cell viability, which is the toxic limit according to ISO standard (ISO 10993-5). (C) Cellular uptake of mPEG-Mn_0.6_Zn_0.4_Fe_2_O_4_ in the upright and inverted configurations in SW480 and Caco-2 cell lines. Intracellular iron content determined by ICP-OES in the SW480 and Caco-2 cells. All data were expressed as mean (ns > 0.05, *p ≤ 0.05, **p ≤ 0.01; *n* ≥ 5).

The interaction of nanoparticles and cells was evaluated in cell lines SW480 and Caco-2. The selection of these cell lines was based on the incidence rate of the cancer type (colorectal adenocarcinoma) and the demographic group (male) from which the cells were derived, both of which are highly represented in the overall population of CRC patients.^49,50^ The concentration-dependent cytotoxicity of pure and mPEGylated Mn_0.6_Zn_0.4_Fe_2_O_4_ and γ-Fe_2_O_3_ was evaluated using the conventional upright configuration (Fig. 6A and B). It has been previously reported that particle sedimentation on the cell surface in the upright configuration results in higher cytotoxicity compared to that in an inverted one.^21^ Thus, the upright cell culture configuration is a more sensitive model for cytotoxicity evaluations. The cell viability decreased with increasing SPION concentration in both cell lines. The cell viability exceeded 80% for both PEGylated SPIONs (in both cell lines) up to 0.4 mg mL^−1^. This is considered non-toxic according to ISO 10993-5.^51^ It is important to note that there was no significant difference (*p* > 0.05) in particle-induced cytotoxicity between Mn_0.6_Zn_0.4_Fe_2_O_4_ and γ-Fe_2_O_3_ at most concentrations investigated. Previously flame-made Zn^2+^ doped ferrites have been reported to exhibit elevated toxicity in Caco-2 cells, even at a low concentration (0.2 mg mL^−1^).^33^ The improved biocompatibility observed in our study is attributed to the hermetic silica coating which serves as a protective barrier between the nanoparticle core and the cells.^52^

An inverted cell configuration (Fig. 1) was implemented to assess nanoparticle–cell interactions as it more accurately reflects particle dynamics in cellular environments.^18–23^ The configuration circumvents the sedimentation of nanoparticles onto the cell surface, which can alter the outcome of nanoparticle uptake. We quantified the iron uptake of SW480 and Caco-2 cells exposed to PEGylated Mn_0.6_Zn_0.4_Fe_2_O_4_ in upright and inverted configurations for comparison. The cellular uptake of iron was comparable in the two cell lines with <4 pg_Fe_ per cell detected by ICP-OES. The low internalization yield of the SPIONs here can be attributed to the densely mPEGylated nanoparticle surface which hinders nanoparticle uptake by the cells.^53,54^ Nanoparticle uptake was slightly higher in the upright configuration than in the inverted one (Fig. 6C). The higher SPION uptake in the upright setup could be attributed to particle sedimentation induced by gravitational forces. This puts the nanoparticles in close contact with the cell surface, in agreement with the literature.^18,55^

### *In vitro* magnetic hyperthermia

The magnetic hyperthermia performance of mPEG-Mn_0.6_Zn_0.4_Fe_2_O_4_ was evaluated in Caco-2 and SW480, in both inverted and upright configurations. While the inverted cell configuration has been applied in literature for uptake and cytotoxicity studies, its role in *in vitro* magnetic hyperthermia assessment has not been explored so far. During AMF exposure, the particles aggregate and sediment on the cells in the upright configuration, potentially enhancing extracellular nanoparticle heating in close proximity to the cell surface. In contrast, the inverted configuration prevents particle sedimentation on the cell surface, resulting in nanoparticle heating being distributed throughout the surrounding medium and thus occurring at a lower particle concentration near the cells. Overall, the cell configuration might thus impact the outcome of magnetic hyperthermia *in vitro*.

**Fig. 7:**
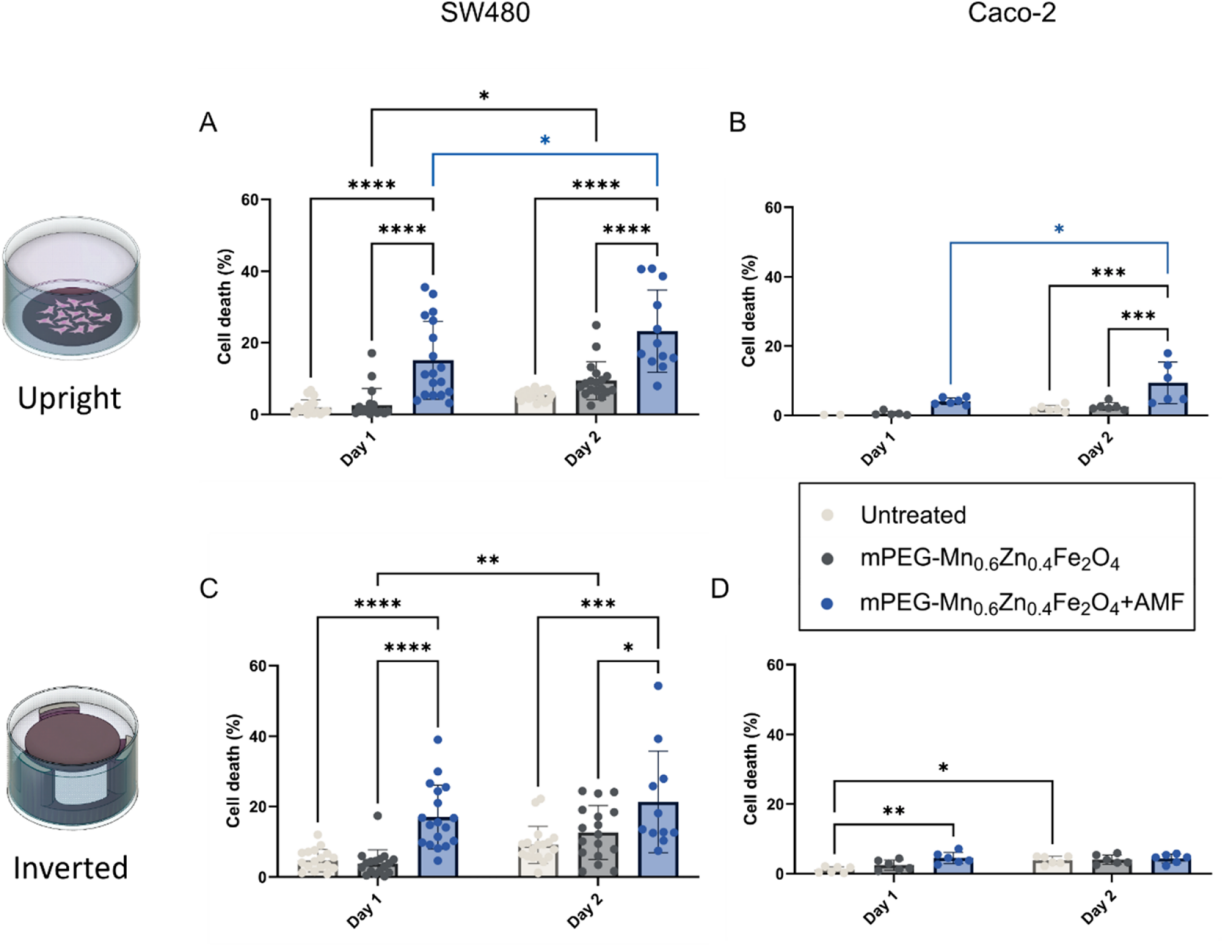
Magnetic hyperthermia outcome in CRC cell lines in upright (A and B) and inverted (C and D) culture configurations with cell culture media (white bars), mPEG-Mn_0.6_Zn_0.4_Fe_2_O_4_ without (grey bars) and with AMF exposure (blue bars), respectively. Cell death of SW480 cells (A and C) and Caco-2 cells (B and D) after exposure to 0.4 mg mL^−1^ mPEG-Mn_0.6_Zn_0.4_Fe_2_O_4_ and AMF for one session of 60 min on day 1 and another 60 min on day 2. All data were expressed as mean ± SD (*p ≤ 0.05, **p ≤ 0.01, ***p ≤ 0.001, ****p ≤ 0.001, *n* ≥ 6).

In this study, two AMF exposures were applied 24 hours apart, as multiple exposures following a single SPION administration is common clinical practice.^56^ Given the low cellular uptake (Fig. 6C), intracellular heating effects can be neglected, but, previous studies have shown effective magnetic hyperthermia treatment also for extracellular nanoparticle localization.^57^Fig. 7 illustrates cell death in both cell lines following one or two AMF exposures. The SPION concentration for the cell studies was selected based on the cytotoxicity assessment (Fig. 6A and B), where the nanoparticles exerted no toxic effects in Caco-2 and SW480 cells at 0.4 mg mL^−1^ (Fig. 6A and B). This ensured that cell death after AMF exposure could primarily be related to magnetic hyperthermia rather than nanoparticle cytotoxicity. This low SPION concentration, restricted the bulk heating efficiency of the suspension and the medium temperature stayed at near physiological levels. Any observed cell death, could thus be primarily attributed to extracellular heating at the nanoparticle level, that can still be effective for magnetic hyperthermia therapy as reported previously.^57,58^

Overall, cell death increased in the AMF-treated group compared to its corresponding controls (either untreated or treated with particles alone) on both day 1 and day 2. A significant (*p* ≤ 0.05) increase in cell death with mPEG-Mn_0.6_Zn_0.4_Fe_2_O_4_ was measured in the upright cell configuration when comparing the first and second AMF exposure (Fig. 7A: day 1 *vs.* day 2). In contrast, no significant difference between the two exposures was observed in the inverted configuration (Fig. 7C). Overall, Caco-2 cells showed less sensitivity to magnetic hyperthermia treatment than the SW480 cells. In the inverted orientation, cell death was only mediated by heating of the surrounding medium, which was insufficient to cause significant cell death, especially for Caco-2 cells on day 2 (Fig. 7D). These findings suggest that the configuration of the cell culture had great impact on the cell death induced by magnetic hyperthermia and particle sedimentation during AMF exposure plays a vital role.

In summary, for a robust preclinical evaluation of SPIONs in magnetic hyperthermia, all parameters, cell line, particle concentration, cellular uptake, experimental configuration, and AMF settings (field strength, duration, number of exposures), must be considered. Future studies could focus on functionalization of the SPIONs with active targeting ligands, to enhance cellular uptake and enable intracellular magnetic hyperthermia.^16^ Thereby, active targeting could also minimize potential thermal damage of healthy tissues by magnetic hyperthermia. Commonly reported upregulated cell surface biomarkers for CRC include vascular endothelial growth factor (VEGF), epidermal growth factor receptor (EGFR) and carcinoembryonic antigen (CEA).^2,59^ An *in vitro* assessment of such nanoparticles would benefit from the use of the inverted cell culture configuration introduced here to evaluate active nanoparticle targeting efficiency and subsequent intracellular magnetic heating.

### *In vivo* magnetic hyperthermia

**Fig. 8:**
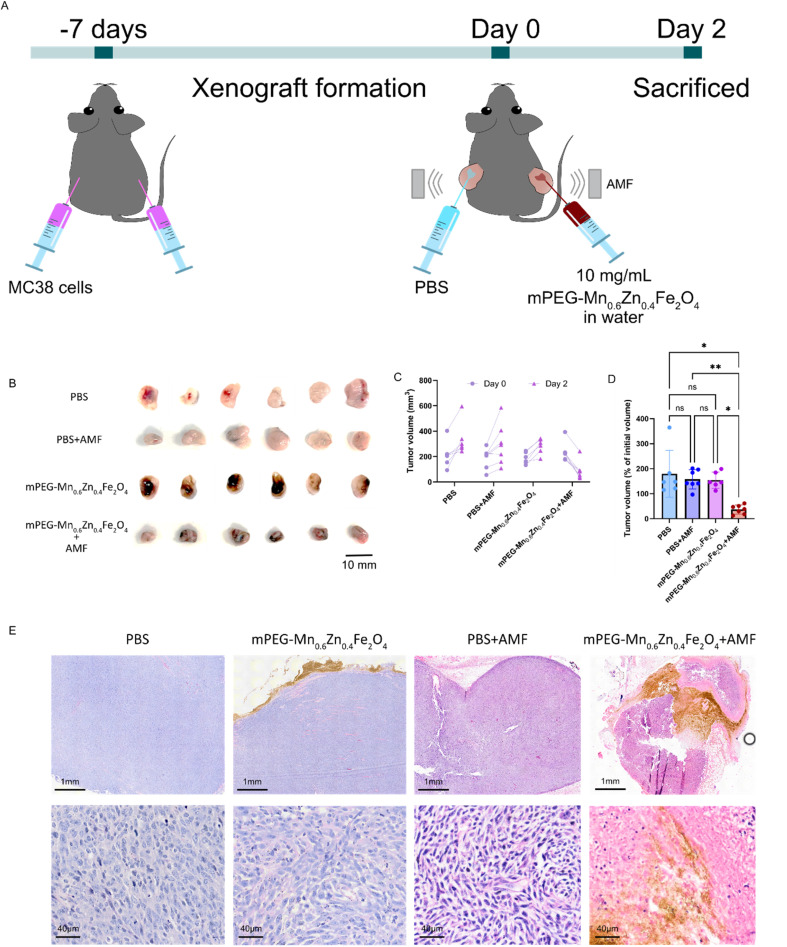
Magnetic hyperthermia outcome in a colorectal tumor xenograft model. (A) Schematic illustration of the *in vivo* magnetic hyperthermia experiment. The xenograft model was established 7 days prior to treatment. On the day of treatment (day 0), either PBS (control) or mPEG-Mn_0.6_Zn_0.4_Fe_2_O_4_ was injected into either tumor of each mouse, followed by a 20-minute AMF exposure. Tumor volumes were measured on day 0 (pre-treatment) and again on day 2 (post-treatment), after which the mice were sacrificed. (B) Representative images of tumors from each treatment group: PBS, PBS + AMF, mPEG-Mn_0.6_Zn_0.4_Fe_2_O_4_ and mPEG-Mn_0.6_Zn_0.4_Fe_2_O_4_ + AMF. (C) Individual tumor volume of all the treatment groups on day 0 and day 2. (D) Percentage tumor volume on day 2 of the initial tumor volume (day 0) of all treatment groups. (E) Representative histological images of tumors treated with PBS, mPEG-Mn_0.6_Zn_0.4_Fe_2_O_4_, PBS + AMF, and mPEG-Mn_0.6_Zn_0.4_Fe_2_O_4_ + AMF. All data were expressed as mean ± SD (*p ≤ 0.05, **p ≤ 0.01, ***p ≤ 0.001, ****p ≤ 0.001; *n* ≥ 6; female = 6, male = 7).

The therapeutic efficacy of magnetic hyperthermia was further evaluated *in vivo* using a colorectal tumor xenograft mouse model bearing bilateral flank tumors. A water suspension of mPEG-Mn_0.6_Zn_0.4_Fe_2_O_4_ or PBS (as control) was injected into either tumor of each mouse, followed by exposure to an AMF for 20 minutes (Fig. 8A). Control mice received similar treatment without the AMF exposure. Tumors were harvested two days later for evaluation.

A SPION concentration of 10 mg mL^−1^ was employed in the *in vivo* study to ensure that a therapeutically relevant temperature under physiological conditions was achieved. In contrast to *in vitro* assays, the *in vivo* environment introduces multiple factors that can attenuate heating efficiency, such as biological heat dissipation, heterogeneous SPION distribution within the tumor, and variability in tumor positioning relative to the magnetic field center. These factors can significantly reduce the overall heating efficiency. *In vitro* heating efficiency of 200 μL of a 10 mg mL^−1^ aqueous mPEG-Mn_0.6_Zn_0.4_Fe_2_O_4_ suspension, corresponding to the injected dose *in vivo*, was assessed under AMF exposure and reached a temperature of 59 °C after 20 minutes (Fig. S7†). In contrast, *in vivo* measurements showed an average surface temperature of 47 °C at the tumor site. These observations underscore the necessity of using higher SPION concentrations *in vivo* to compensate for physiological heat loss and to achieve therapeutic intertumoral temperatures.

Notably, tumors treated with magnetic hyperthermia appeared visibly smaller than those in all other treatment groups (Fig. 8B). Quantitative analysis of individual tumor volumes (Fig. 8C), revealed that only the magnetic hyperthermia group exhibited a reduction in tumor size over the two-day period, whereas tumors in the other groups (PBS, PBS + AMF, and mPEG-Mn_0.6_Zn_0.4_Fe_2_O_4_ alone) continued to grow. When tumor volumes on day 2 were normalized to their initial sizes on day 0, the mPEG-Mn_0.6_Zn_0.4_Fe_2_O_4_ exposed to the AMF group showed a significant reduction to 37% of its original size. In contrast, tumor volumes increased to 179% in the PBS group, 157% in the PBS with AMF exposure group, and 154% in the mPEG-Mn_0.6_Zn_0.4_Fe_2_O_4_ group (Fig. 8D).

Histological analysis further confirmed the therapeutic effect of magnetic hyperthermia (Fig. 8E). Tumors treated with mPEG-Mn_0.6_Zn_0.4_Fe_2_O_4_ and exposed to AMF showed characteristic signs of necrosis, including predominant pink staining, absence of nuclei (karyolysis), and the presence of condensed nuclei (pyknosis), particularly in areas adjacent to nanoparticles (the brown stains). These tumors exhibited markedly reduced nuclear staining and widespread necrotic regions, composed of cellular material and extracellular matrix. No histological alterations were observed in tumors treated with PBS, mPEG-SPIONs alone, or PBS + AMF, indicating that neither the particles nor AMF exposure alone caused tissue damage. Overall, these results demonstrate that a single 20-minute magnetic hyperthermia exposure with the mPEG-Mn_0.6_Zn_0.4_Fe_2_O_4_ nanoparticles can induce substantial tumor regression, underscoring their potential as an effective therapeutic strategy for CRC.

## Conclusion

In this study, we manufactured superparamagnetic silica-coated Mn_0.6_Zn_0.4_Fe_2_O_4_ nanoparticles by FSP and functionalized their surface with a high density of PEG chains to render them physically stable in biorelevant colonic microenvironments. We also evaluated their potential as orally administered theranostic agents for CRC. The mPEG-Mn_0.6_Zn_0.4_Fe_2_O_4_ exhibited excellent and consistent magnetic heating performance in biorelevant media, including colonic mucus, gastrointestinal luminal fluids, and tissue, as well as enhanced *T*_2_ contrast compared to γ-Fe_2_O_3_. The hyperthermia performance of the particles was further tested in SW480 and Caco-2, cell lines typically used during preclinical evaluation of CRC therapies. Inverted and upright culture configurations were assessed. To the best of our knowledge, this study is the first demonstration of the impact of cell culture configuration on *in vitro* assessment of magnetic hyperthermia. Hyperthermia treatment in the traditional upright configuration showed a marked increase in cell death compared to the inverted configuration. These findings highlight the importance of a systematic and accurate *in vitro* evaluation of nanoparticles to circumvent false positive results that could hamper the clinical translation of promising theranostic agents for cancer treatment. *In vivo* evaluation revealed a significant reduction in tumor size two days after SPION injection and AMF exposure. In conclusion, mPEG-Mn_0.6_Zn_0.4_Fe_2_O_4_ demonstrated strong potential for theranostic magnetic hyperthermia applications both *in vitro* and *in vivo*.

## Author contributions

Y. Z.: conceptualization, formal analysis, funding acquisition, investigation, methodology, visualization, writing – original draft, writing – review & editing. C. P.: formal analysis, investigation, methodology, writing – review & editing. Q. C.: formal analysis, investigation, methodology, visualization, writing – original draft, writing – review & editing. A. M.: investigation, writing – review & editing. S. R. A.: investigation, writing – review & editing. T. S.: investigation, writing – review & editing. V. K.: conceptualization, funding acquisition, supervision, methodology, project administration, resources, writing – original draft, writing – review & editing. A. T.: conceptualization, funding acquisition, supervision, methodology, project administration, resources, writing – original draft, writing – review & editing.

## Conflicts of interest

There are no conflicts to declare.

## Supplementary Material

NA-OLF-D5NA00603A-s001

## Data Availability

Data for this article are available at SciLifeLab Data Repository at https://doi.org/10.17044/scilifelab.27688668.
