# Supplementary material for: Flame-made nanoparticles for magnetic hyperthermia and MRI in colorectal cancer theranostics
Source: Nanoscale Adv. 2025 Jul 16;7(17):5284–99. doi: 10.1039/d5na00603a (PMC12281419; doi:10.1039/d5na00603a)
Supplement: NA-007-D5NA00603A-s001 [file NA-007-D5NA00603A-s001.pdf]

Supplementary Information (SI) for Nanoscale Advances.
This journal is © The Royal Society of Chemistry 2025

Supporting Information for

Flame-made nanoparticles for magnetic hyperthermia and MRI theranostics in colorectal cancer

[1]

*Yuming Zhang[1], Christina Paraskeva[2], Qianying Chen[1], Anano Maisuradze[1], Shaquib Rahman Ansari[1], Tapati Sarkar[3], Vasiliki Koliaraki[2] and Alexandra Teleki[1]**

[1]Department of Pharmacy, Science for Life Laboratory, Uppsala University, 75123 Uppsala, Sweden

[2]3Institute for Fundamental Biomedical Research, Biomedical Sciences Research Center 'Alexander Fleming', 16672 Vari, Greece

[3]Department of Materials Science and Engineering, Uppsala University, Box 35, 75103 Uppsala, Sweden

*Corresponding author: alexandra.teleki@scilifelab.uu.se

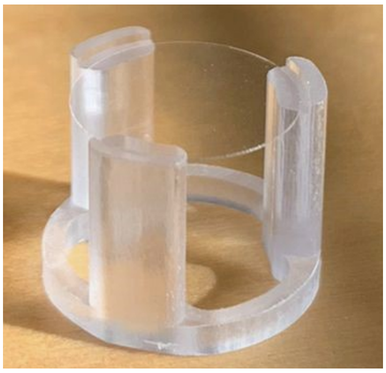

Figure S1. The inverted cell culture configuration. The coverslip was placed on the top of the insert thereby forcing cells to face downwards.

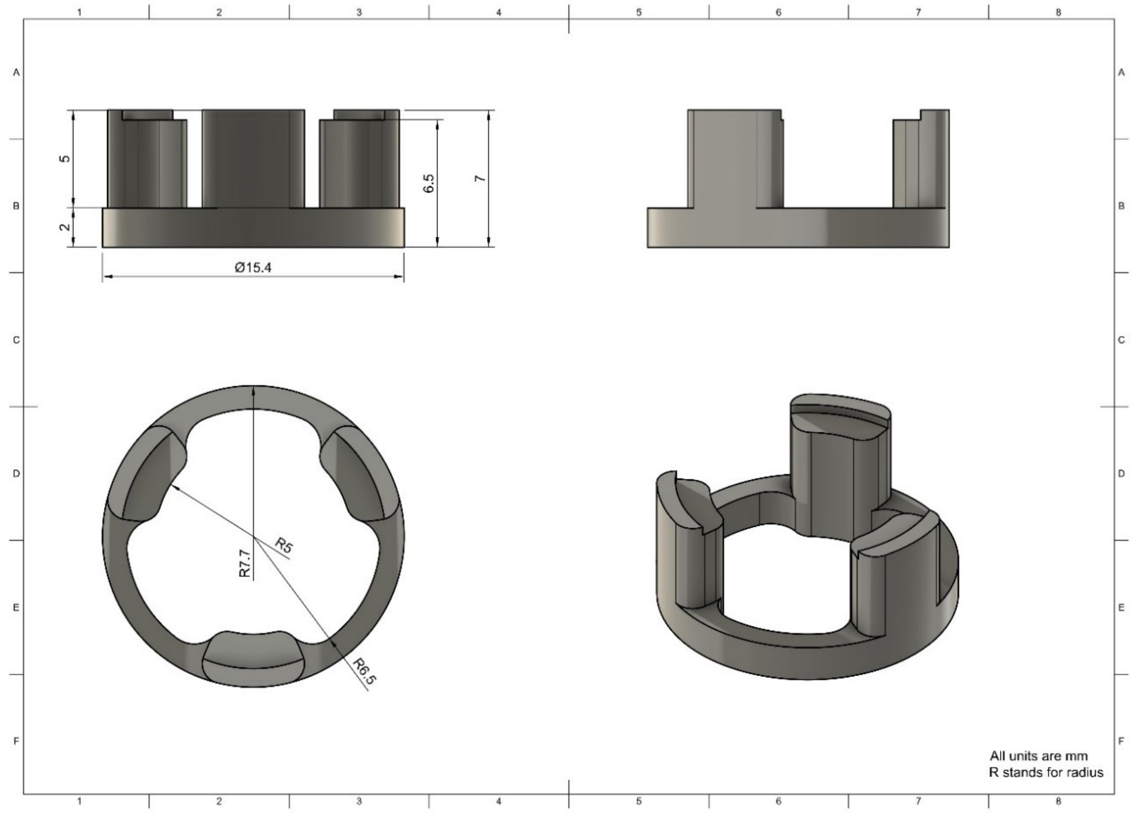

Figure S2. Graphical design of the inverted insert.

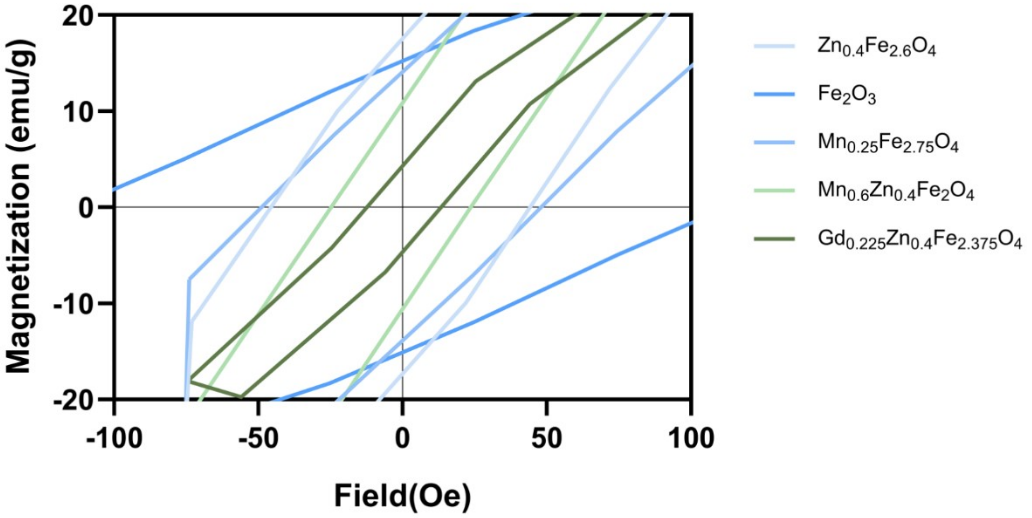

Figure S3. Magnification of the magnetization curves at ±100 Oe from Figure 2D for silica-coated γ-Fe_2O_3, $Mn_{0.25}Fe_{2.75}O_4$, $Zn_{0.4}Fe_{2.6}O_4$, $Mn_{0.6}Zn_{0.4}Fe_2O_4$ and $Gd_{0.225}Zn_{0.4}Fe_{2.375}O_4$.

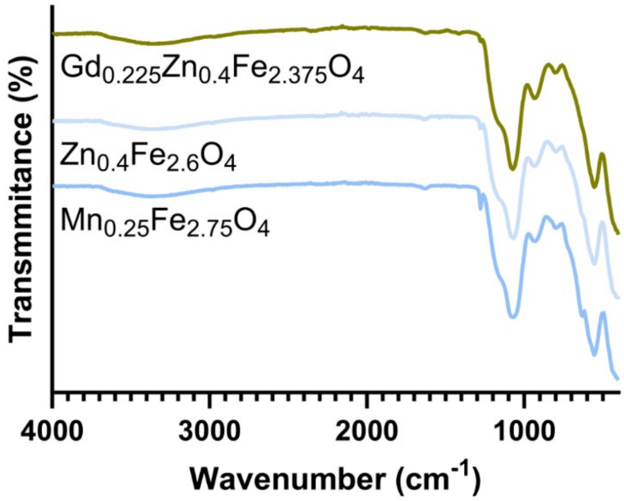

Figure S4. FTIR spectra of silica-coated $Mn_{0.25}Fe_{2.75}O_4$, $Zn_{0.4}Fe_{2.6}O_4$, and $Gd_{0.225}Zn_{0.4}Fe_{2.375}O_4$.

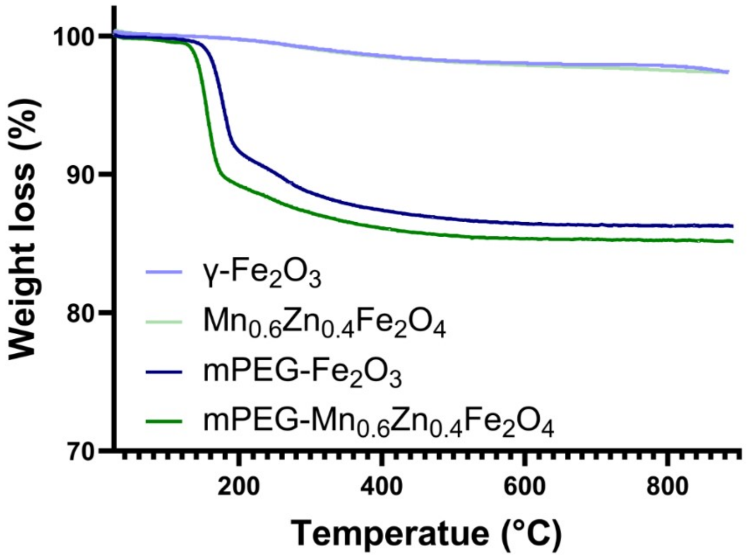

Figure S5. Thermal decomposition curve of SPIONs before and after mPEGylation.

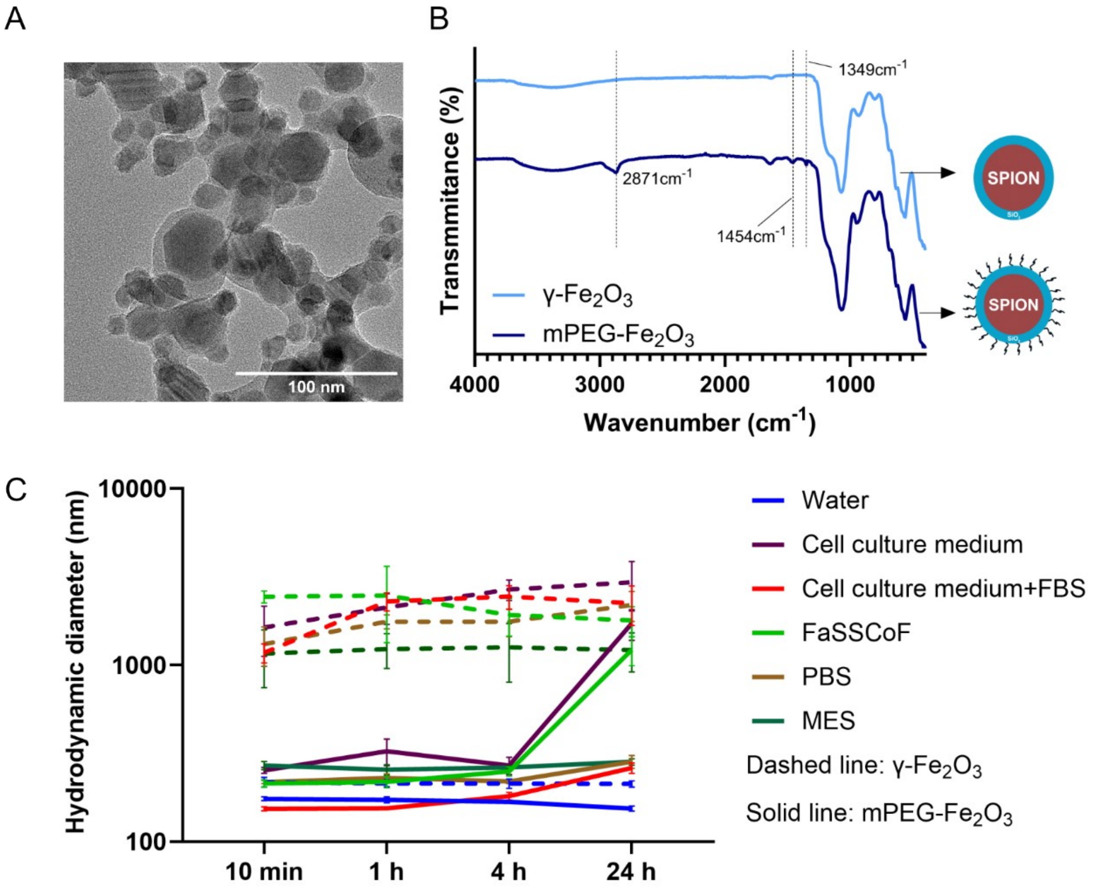

Figure S6. Characterization of the mPEGylated γ-Fe_2O_3 and its colloidal stability in biological fluids. **(A)** Transmission electron microscope image of the mPEG-Fe_2O_3 **(B)** FTIR spectra before and after surface PEGylation. Light blue line: γ-Fe_2O_3. Dark blue line: mPEG-Fe_2O_3. **(C)** Characterization of particle suspension stability in biological fluids. Changes in hydrodynamic diameter of the γ-Fe_2O_3 (1 mg/mL) before and after mPEGylation over 24 h in: in water (blue), cell culture medium (dark red), cell culture medium with fetal bovine serum (FBS; bright red), simulated fasted-state colonic fluid (FaSSCoF; green), phosphate-buffered saline (PBS; brown), and 2-(N-morpholino)ethanesulfonic acid (MES; dark green). Dashed line: as prepared. Solid line: mPEGylated. All data are expressed as mean ± SD (n=3).

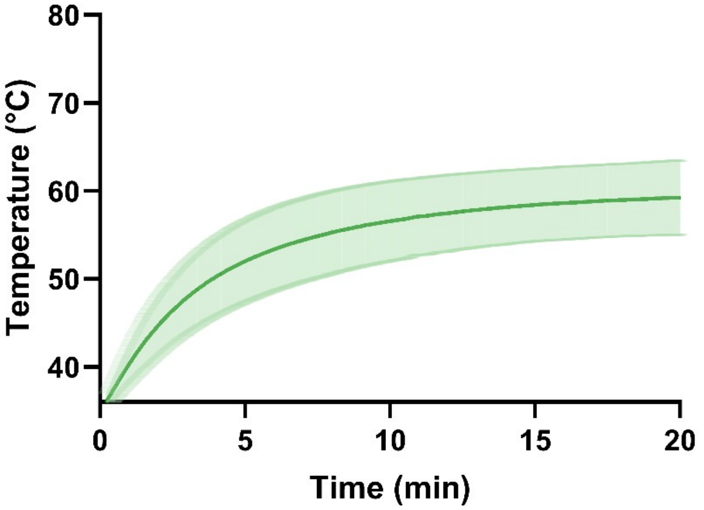

Figure S7. *In vitro* heating performance of 200 µL of 10 mg/mL of mPEG-$Mn_{0.6}Zn_{0.4}Fe_2O_4$ water suspension. Data is expressed as mean ± standard deviation (n = 6).

Table S1. PEG content and zeta potential before and after surface PEGylation. Data expressed as mean ± SD (n=9 for mPEG content and n=3 for zeta potential).

SPIONs	Zeta potential (mV)	mPEG content w/w (%)	mPEG surface coverage-density σ (PEG/nm^2)	mPEG footprint, FP (nm^2/chain of PEG)	Distance between anchored mPEG chains on the surface (nm)	Unperturbed layer thickness (nm)
γ-Fe_2O_3	-52.6 ± 1.9					
mPEG-Fe_2O_3	-42.2 ± 2.1	11.0 ± 0.7	0.30 ± 0.02	3.4 ± 0.2	2.1 ± 0.1	12.2 ± 0.3
$Mn_{0.6}Zn_{0.4}Fe_2O_4$	-34.2 ± 1.6					
mPEG-$Mn_{0.6}Zn_{0.4}Fe_2O_4$	-29.5 ± 0.5	11.7 ± 1.3	0.32 ± 0.03	3.2 ± 0.4	2.0 ± 0.1	12.4 ± 0.5
